# An EGFR‐targeting antibody–drug conjugate LR004‐VC‐MMAE: potential in esophageal squamous cell carcinoma and other malignancies

**DOI:** 10.1002/1878-0261.12400

**Published:** 2018-11-15

**Authors:** Xin‐yue Hu, Rong Wang, Jie Jin, Xiu‐jun Liu, A‐long Cui, Lian‐qi Sun, Yan‐ping Li, Yi Li, Yu‐cheng Wang, Yong‐su Zhen, Qing‐fang Miao, Zhuo‐rong Li

**Affiliations:** ^1^ Institute of Medicinal Biotechnology Chinese Academy of Medical Sciences Peking Union Medical College Beijing China

**Keywords:** antitumor activity, EGFR, ESCC, LR004, LR004‐VC‐MMAE, preparation

## Abstract

Epidermal growth factor receptor (EGFR) is a rational target for cancer therapy, because its overexpression plays an important oncogenic role in a variety of solid tumors; however, EGFR‐targeted antibody–drug conjugate (ADC) therapy for esophageal squamous cell carcinoma (ESCC) is exceedingly rare. LR004 is a novel anti‐EGFR antibody with the advantages of improved safety and fewer hypersensitivity reactions. It may be of great value as a carrier in ADCs with high binding affinity and internalization ability. Here, we prepared an EGFR‐targeting ADC, LR004‐VC‐MMAE, and evaluated its antitumor activities against ESCC and EGFR‐positive cells. LR004 was covalently conjugated with monomethyl auristatin E (MMAE) via a VC linker by antibody interchain disulfide bond reduction. VC‐MMAE was conjugated with LR004 with approximately 4.0 MMAE molecules per ADC. LR004‐VC‐MMAE showed a potent antitumor effect against ESCC and other EGFR‐positive cells with IC_50_ values of nM concentrations *in vitro*. The *in vivo* antitumor effects of LR004‐VC‐MMAE were investigated in ESCC KYSE520 and A431 xenograft nude mice models. Significant activity was seen at 5 mg·kg^−1^, and complete tumor regression was observed at 15 mg·kg^−1^ in the KYSE520 xenograft nude mice after four injections, while the naked antibody LR004 had little effect on inhibiting tumor growth. Similar promising results were obtained in the A431 models. In addition, the tumors also remained responsive to LR004‐VC‐MMAE for large tumor experiments (tumor volume 400–500 mm^3^). The study results demonstrated that LR004‐VC‐MMAE could be a potential therapeutic agent for ESCC and other EGFR‐expressing malignancies. We also evaluated PK profile of LR004‐VC‐MMAE ADC in the mice model, which would provide qualitative guiding significance for the further research.

**Abbreviations**
ADC: antibody–drug conjugate
ALCL: anaplastic large‐cell lymphoma
BrdUrd: bromodeoxyuridine
CCK‐8: cell counting Kit‐8
DAPI: 4′, 6‐diamidino‐2‐phenylindole
DAR: average drug–antibody ratio
EGFR: epidermal growth factor receptor
ESCC: esophageal squamous cell carcinoma
LAMP‐1: lysosomal‐associated membrane protein 1
MMAE: monomethyl auristatin E
NSCLC: non‐small‐cell lung cancer
TMB: 3,5,3′,5′‐tetramethylbenzidine

## 1 Introduction

The transmembrane glycoprotein epidermal growth factor receptor (EGFR) is a member of the EGFR family of receptor tyrosine kinases (TK). Components of the extracellular transmembrane and intracellular tyrosine kinase domains correlate with cell proliferation, progression, and metastasis. EGFR is a rational target for cancer therapy, because its overexpression plays an important oncogenic role in a variety of solid tumors, such as head and neck, breast, lung, and colorectal cancer (Ang *et al*., 2002; Reis‐Filho *et al*., 2005; Selvaggi *et al*., 2004; Repetto *et al*., 2005). Currently, there are two distinct therapeutic strategies employed for EGFR‐targeted cancer therapy: One is monoclonal antibodies and the other is small‐molecule tyrosine kinase inhibitors (TKIs). Anti‐EGFR antibodies exert antitumor effects by binding the receptor at the cell surface to interfere with ligand binding, which leads to the inhibition of its downstream signaling pathway. The approved naked antibodies (i.e., cetuximab, nimotuzumab, panitumumab, and necitumumab) for EGFR demonstrate their therapeutic utility in malignancies but are often used in combination with chemotherapy drugs to achieve significant clinical efficacy (Xiong *et al*., 2004; Kim *et al*., 2013). Furthermore, since the use of an antibody as a single agent is suboptimal, an antibody–drug conjugate (ADC) is one of the potential strategies to increase the antitumor activity of an antibody. In EGFR‐targeted therapy, several ADCs have entered clinical trials. AVID‐100, which is composed of the maytansinoid attached to an anti‐EGFR antibody, was developed to treat epithelial tumor patients in phase I/II (O'Connor‐McCourt *et al*., 2016; Tolcher *et al*., 2018). ABT‐414, which is composed of the monomethyl auristatin F attached to an anti‐EGFRvIII antibody via a cleavable linker, has shown significant efficacy against tumors expressing amplified EGFR and EGFRvIII in phase III (van den Bent *et al*., 2017).

Several solid malignancies have been reported to be treated by EGFR‐targeted ADCs (Ojima *et al*., 2002; Patra *et al*., 2008; Phillips *et al*., 2016). However, EGFR‐targeted ADC therapy for esophageal squamous cell carcinoma (ESCC) is exceedingly rare. Esophageal cancer is the 7th most common cancer worldwide and the 6th leading cause of cancer‐related deaths (Global Burden of Disease Cancer Collaboration *et al*., 2017). ESCC is a major subtype of esophageal cancer, with most patients presenting with advanced‐stage disease and consequently having a poor prognosis. Despite a general recognition of the high incidence and lethality of this disease, the development of novel therapies for patients with ESCC seems to lag behind other solid malignancies. Generally, EGFR overexpression is recognized as an indicator of poor prognosis in ESCC. Several studies demonstrate that high EGFR expression occurs in 70–88% of patients with ESCC (Salomon *et al*., 1995; Nicholson *et al*., 2001). Complete surgical resection is one of the most important standard treatments, but the 5‐year survival rate is only 15–25% (Lin *et al*., 2015). Currently, there are no drugs showing therapeutic effect that can be administered to treat ESCC.

LR004 is an anti‐EGFR antibody, with the advantages of improved safety and fewer hypersensitivity reactions (Eric, 2016). The sialic acid of LR004 is N‐acetylneuraminic acid (NANA), which is considered to be more humanlike, and the glycoengineering modification of LR004 is less immunoreactive. In addition, LR004 also demonstrates a long serum half‐life and high thermostability. When used as a single agent, LR004 hampers the growth of several tumor xenografts, such as epidermoid carcinoma (A431), colon cancer (GEO), and breast cancer (MDA‐MB‐468). LR004 is also named SYN004, which has been conducting in patients with solid tumors in phase I (Papageorgiou *et al*., 2016; Synermore Biologics Co., Ltd, 2017; Riley *et al*., 2018). On the basis of the properties of LR004, we anticipated that LR004 conjugated to potent antitubulin drugs would be most effective in ESCC and other solid tumor cells with high levels of EGFR expression.

Here, we initially synthesized an ADC, in which the cytotoxic drug monomethyl auristatin E (MMAE) was conjugated to LR004. Then, we screened various types of tumor cells with different EGFR expression levels and assessed the antitumor activities of LR004‐VC‐MMAE *in vitro*. Moreover, we mainly evaluated the functional characterization and antitumor activities of LR004‐VC‐MMAE in ESCC cell lines and A431 cells *in vitro* and *in vivo*. Based on these findings, the ADC of LR004‐VC‐MMAE could be an enlightened EGFR‐targeted therapy for ESCC and other tumors, with its potent effectiveness. The preliminary PK profile would provide the basis for further studies of LR004‐VC‐MMAE with cancer.

## 2 Materials and methods

### 2.1 Cell lines and reagents

Human ESCC (KYSE150), epidermoid carcinoma (A431), and non‐small‐cell lung cancer (NSCLC; A549, NCI‐H1975, HCC827) cell lines were obtained from the Chinese Academy of Sciences (Shanghai, China). Human anaplastic large‐cell lymphoma (ALCL; Karpas 299) cell lines were purchased from BIOPIKE (Beijing, China). Human ESCC (KYSE520) cell lines were obtained from Creative Bioarray, Inc. The breast (MDA‐MB‐468, MCF‐7) cancer cell lines and pancreatic (AsPC‐1) cancer cell lines were provided by our laboratory. The KYSE520 and KYSE150 cells were cultured in RPMI‐1640/F12. The A431, MDA‐MB‐468, A549, and MCF‐7 cells were maintained in DMEM supplemented with 10% FBS. The HCC‐827, NCI‐H1975, AsPC‐1, and Karpas 299 cells were cultured in RPMI‐1640 supplemented with 10% FBS. All the cells were cultured in an incubator and were maintained at 37 °C with 5% CO_2_. The LR004 antibody was kindly provided from Shandong Xinhua Pharmaceutical Co., Ltd (Zibo, Shandong, China), and was prepared by Shenzhen Lonn Ryonn Pharmaceutical (Shenzhen, Guangdong, China). Rituximab was purchased from the Cancer Hospital, Chinese Academy of Medical Science. The anti‐CD30 antibody was provided by the Oncology Department of the Institute of Medicinal Biotechnology. The preparation and characterization of rituximab‐VC‐MMAE (Fig. S3A) and anti‐CD30‐VC‐MMAE (Fig. S3B) were similar to LR004‐VC‐MMAE.

### 2.2 Animals

BALB/c nude mice (6–8 weeks old, 18–20 g, female) were purchased from Beijing SPF Biotechnology Co., Ltd (Beijing, China), and were housed at a controlled temperature of 25 °C under pathogen‐free conditions in a humidity‐controlled environment. The mice were acclimated for 1 week prior to the commencement of the experiments, and all the experiments were approved by the Animal Care and Use Committee of People's Republic of China.

### 2.3 Preparation of LR004‐VC‐MMAE

**Figure 1:**
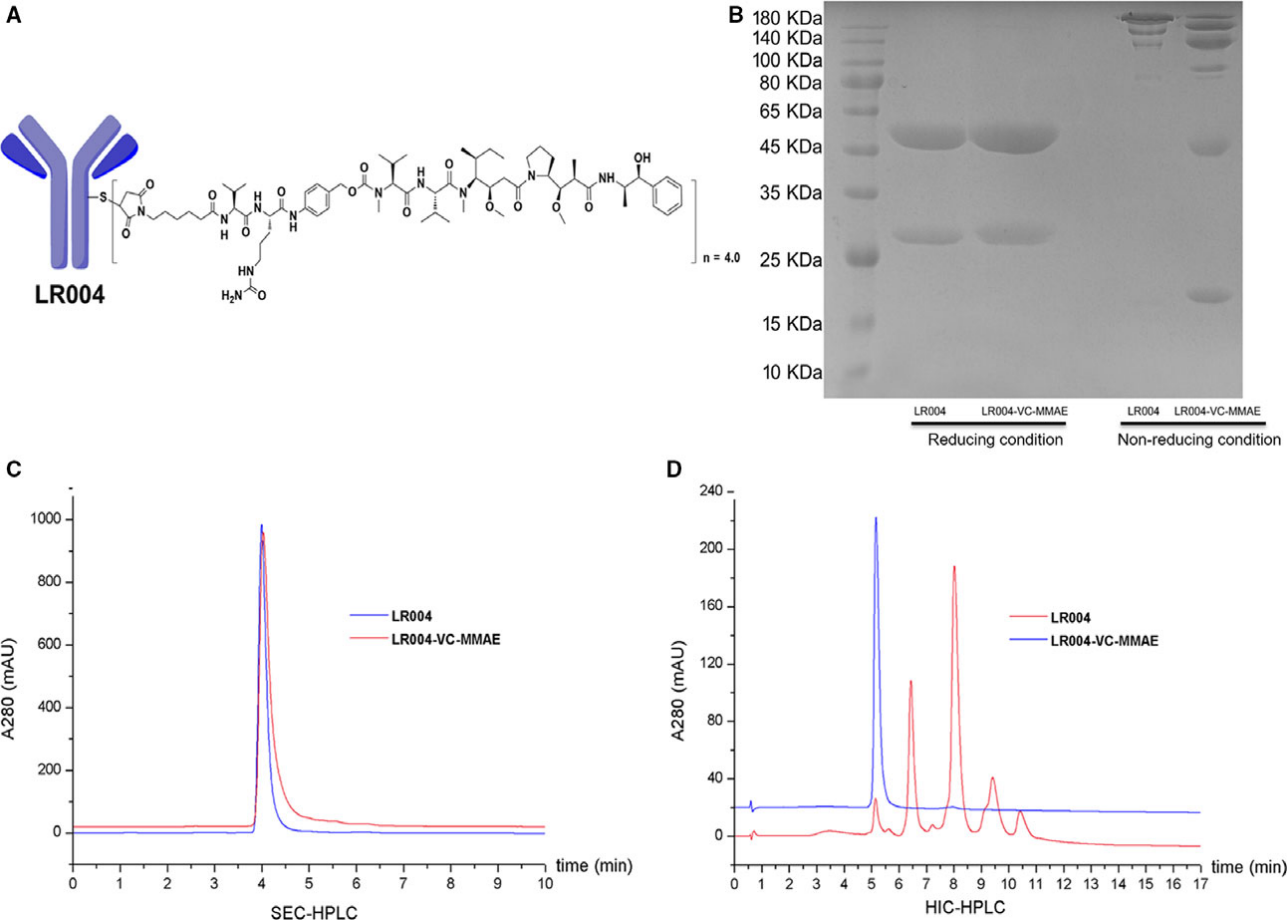
Characterization of LR004 and LR004‐VC‐MMAE. (A) The structure of LR004‐VC‐MMAE. (B) SDS/PAGE analysis of LR004 and LR004‐VC‐MMAE under the reducing and nonreducing conditions (gradient 4–12%). (C) SEC‐HPLC analysis of LR004 and LR004‐VC‐MMAE. LR004, RT (retention time) = 3.998 s; LR004‐VC‐MMAE, RT (retention time) = 4.031 s. (D) HIC analysis of LR004 and LR004‐VC‐MMAE. The HIC‐HPLC spectrum of LR004‐VC‐MMAE displays five major peaks, corresponding to zero, two, four, six, and eight drugs per antibody. The average DAR of LR004‐VC‐MMAE is approximately 4.0 after integration of the observed peaks.

LR004 in a 0.025 mm borate and sodium chloride buffer (containing 1 mm DTPA, pH = 8) was mixed with approximately threefold TCEP and was stirred for 2 h at 37 °C under the protection of nitrogen. The reaction system was quickly dropped over eightfold for VC‐MMAE and was incubated for 1 h on ice, and 20‐fold excess of cysteine was added over the drug linker to extinguish the reaction. Finally, the ADC product (Fig. 1A) was purified by elution through Sephadex G‐25 equilibrated in PBS and concentrated by centrifugal ultrafiltration. The conjugate was filtered through a 0.2‐μm filter under sterile conditions and stored at −80 °C for analysis and testing.

### 2.4 Hydrophobic interaction chromatography (HIC) analysis

The characterization of LR004‐VC‐MMAE was analyzed using HIC as follows: 1200 HPLC (Agilent Technologies, Beijing, China); TSKgel Butyl‐NPR column (4.6 × 35 mm, particle size 2.5 μm; TOSOH, Tokyo, Japan); solvent A, 1.5 mol·L^−1^ ammonium sulfate and 25 mm phosphate (pH = 6.95); solvent B, 75% (V/V) 25 mm phosphate, 25% (V/V) isopropanol (pH = 6.95); the gradient was 100% A to 100% B over 15 min; 0.5 mL·min^−1^ flow rate; column temperature was 25 °C; UV detection wavelength was 280 nm.

### 2.5 Size‐exclusion chromatography (SEC) analysis

Size‐exclusion chromatography analyses were used to detect purity of LR004 and LR004‐VC‐MMAE. 1200 HPLC (Agilent Technologies); Acquity UPLC protein BEH SEC column (4.6 × 150 mm, particle size 1.7 μm, Aperture 200 Å; Waters); mobile phase 100 mm ammonium acetate (pH = 8); 0.3 mL·min^−1^ flow rate; column temperature was 30 °C; UV detection wavelength was 280 nm.

### 2.6 Binding affinity of LR004 and LR004‐VC‐MMAE *in vitro*

#### 2.6.1 ELISA

High‐binding 96‐well plates were coated with 1 μg·mL^−1^ recombinant EGFR protein (ACRO Biosystems, Beijing, China) at 4 °C overnight. After blocking for 1 h at 37 °C with 1% BSA in PBS, various concentrations of LR004 and LR004‐VC‐MMAE were incubated at 37 °C for 1 h. The wells were washed three times with PBST and were incubated with an anti‐human IgG (Fab‐specific)–alkaline phosphatase–goat at 37 °C for 2 h. The 3,5,3′,5′‐tetramethylbenzidine (TMB) was added to the wells for color development, and the reaction was stopped by 2 m H_2_SO_4_. The optical density (OD) was read at 450 nm using a microplate reader (Thermo Fisher Scientific, Franklin, MA, USA).

#### 2.6.2 Flow cytometric analysis

A total of 3 × 10^5^ cells were incubated with various concentrations (3, 1, 0.3, 0.1, 0.03, 0.01, and 0.003 μg·mL^−1^) of LR004 and LR004‐VC‐MMAE in PBS/1% FBS for 1 h at 4 °C. The cells were then washed three times with PBS and incubated with 1 : 100 FITC‐labeled goat anti‐human IgG for 1 h at 4 °C. The labeled cells were washed, resuspended in PBS, and analyzed by a FACSCalibur (BD Biosciences, San Jose, CA, USA).

#### 2.6.3 Biacore study

The CM5 sensor chip was pre‐immobilized with LR004 and LR004‐VC‐MMAE at a concentration of 1 μg·mL^−1^. The antigen EGFR (ACRO Biosystems) was injected at a flow of 30 μL·min^−1^ at concentrations ranging from 15.625 to 1000 ng·mL^−1^ in HEPES buffer. The sensor surface was regenerated between each binding reaction with 3 m MgCl_2_. The biacore t200 evaluation software was used to determine the rate constants *k*_on_ and *k*_off_.

### 2.7 Confocal analysis for intracellular localization

The KYSE520 cells were seeded at a density of 1 × 10^4^ cells·well^−1^ and incubated for 24 h; they were then treated with 5 μg·mL^−1^ LR004 and LR004‐VC‐MMAE for 30 min at 4 °C or for 0.5 and 10 h at 37 °C. After washing the wells with PBS, the cells were exposed on the chamber coverslip by the cytospin, fixed with 4% paraformaldehyde for 10 min, and permeabilized with 0.2% Triton X‐100 for 5 min. LR004 and LR004‐VC‐MMAE were detected with an Alexa Fluor 488‐labeled goat anti‐human IgG (H+L) antibody. The lysosomes were labeled with a lysosomal‐associated membrane protein 1 (LAMP‐1) antibody followed by an Alexa Fluor 555‐labeled goat anti‐rabbit IgG (H+L) antibody. The cell nuclei were stained with 4′, 6‐diamidino‐2‐phenylindole (DAPI). Fluorescence images were acquired with the laser scanning confocal microscope (ZEISS LSM 710, Oberkochen, Germany).

### 2.8 Flow cytometry for apoptosis and cell cycle arrest analysis

To evaluate cell apoptosis in various types of cells, cells were seeded at a density of 2 × 10^5 ^cells/well and exposed to LR004‐VC‐MMAE with various concentrations (100, 30, and 10 μg·mL^−1^) for 24 h; the control group was treated with medium alone. Apoptosis and cell death were detected using an Annexin V‐FITC Apoptosis Kit (Dojindo, Japan) and propidium iodide (PI) staining by a FACSCalibur (BD Biosciences). For the cell cycle position analysis after drug exposure, the cells were treated as described above and allowed to incorporate bromodeoxyuridine (BrdUrd; Beyotime Biotechnology, Shanghai, China) for 20 min. Nascent DNA synthesis was detected with anti‐BrdUrd FITC, and total DNA content was detected with PI. The cell cycle position and apoptosis analyses were measured by a FACSCalibur (BD Biosciences).

### 2.9 Evaluation of LR004‐VC‐MMAE for tumor cell killing *in vitro*

The effects of LR004, LR004‐VC‐MMAE, and MMAE on cell growth were analyzed by the cell counting kit‐8 (CCK‐8) assay. The cells were plated at 1 × 10^3^ to 3 × 10^3^ in 100 μL complete medium into 96‐well plates. After an overnight incubation at 37 °C, the cells were added at various concentrations (1, 0.3, 0.1, 0.03, 0.01, 0.003, and 0.001 μg·mL^−1^) with 100 μL medium for 48 h, and 20 μL of the CCK‐8 reagent was added to measure cell viability. The absorbance was measured at 450 nm by a microplate reader, and the cell viability ratio (%) was calculated using the following formula: [(*A* sample‐*A* blank)/(*A* control‐*A* blank)] × 100%. The 50% inhibitory concentration (IC_50_) of the samples was calculated by spss software (IBM SPSS, Chicago, IL, USA). The data are presented as the mean ± SD from three independent experimental titrations.

### 2.10 *In vivo* fluorescence imaging experiment

The *in vivo* fluorescence imaging experiments were investigated using the KYSE520 nude mice xenograft model. LR004, LR004‐VC‐MMAE, and rituximab‐VC‐MMAE were labeled with DyLight 680 according to the manufacturer's instructions (Dylight 680 Antibody Labeling Kit, Thermo Fisher Scientific). When the tumor size reached approximately 300 mm^3^, mice in the three DyLight 680‐labeled groups were injected via the tail veins with the dose of 20 mg·kg^−1^ each. The mice were imaged under anesthesia at the indicated time points after the injection using the IVIS 200 imaging system. The data were analyzed using living image software (Xenogen, Alameda, CA, USA).

### 2.11 Evaluation of LR004‐VC‐MMAE for antitumor efficacy *in vivo*

In the ESCC model, the BALB/c nude mice were injected subcutaneously in the right armpit with 5 × 10^6^/100 μL KYSE520 tumor cells. The treatment was initiated on the ninth day, when the mean tumor size in each group of eight animals was approximately 100 mm^3^. The mice were treated with various doses of LR004‐VC‐MMAE (5, 10, 15 mg·kg^−1^), LR004 (15 mg·kg^−1^), and rituximab‐VC‐MMAE (15 mg·kg^−1^) every 4 days for a total of four injections. In the A431 model, 5 × 10^6^/100 μL A431 tumor cells were implanted subcutaneously into the right armpit of the BALB/c nude mice. Therapy was started on the seventh day, when the tumor volumes reached approximately 100 mm^3^; the mice were randomly divided into 7 groups (*n* = 6 per group) and were treated with LR004 (15 mg·kg^−1^), LR004‐VC‐MMAE (1, 5, 10, 15 mg·kg^−1^), and MMAE (0.3 mg·kg^−1^) every 4 days for a total of six injections.

Meanwhile, a large tumor group for the A431 tumor xenograft model was established on the tenth day, when the tumor volume reached a size of 400–500 mm^3^; the mice were given an intravenous injection (LR004‐VC‐MMAE 15 mg·kg^−1^) every 4 days for a total of six injections. The tumors were measured 2–3 times per week using a caliper measurement. Tumor volumes were calculated according to the formula: *V* = *L*×*W* ^2^/2, where L is the longest diameter of the tumor and *W* is the shortest diameter perpendicular to *L*. The tumor growth inhibition rate was calculated as [1‐tumor volume (treated)/tumor volume (control)] × 100%. At the end of experiment, the mice were euthanized, and various organs and tumors were harvested and fixed in 10% formalin for histopathological examination (H&E).

### 2.12 Pharmacokinetic studies in nude mice model

#### 2.12.1 ELISA quantification of total antibody in nude mice

Forty nude mice were divided as four mice per group (per time point). Mice were dosed as single intravenous injections (15 mg·kg^−1^). Whole‐blood sample was collected from cardiac puncture at selected times for up to 9 days (0, 0.5, 1, 2, 8, 24, 48, 72, 120, and 216 h). After blood collection, blood was then centrifuged and the plasma fraction was separated and collected in 1 mL 96‐well plate format and stored at −80 °C until analysis. The concentration of total antibody was analyzed by ELISA method. The 96‐well plates were coated with 1 μg·mL^−1^ recombinant EGFR protein (ACRO Biosystems) at 4 °C overnight. After blocking for 1 h at 37 °C with 1% BSA in PBS, samples were added after 40 000‐fold dilution with ELISA buffer (calibration standard: 40–0.625 ng·mL^−1^). The samples were incubated for 1 h at 37 °C, and then, the wells were washed three times with PBST and were incubated with an anti‐human IgG (Fab‐specific)–alkaline phosphatase–goat at 37 °C for 2 h. The TMB was added to the wells for color development, and the reaction was stopped by 2 m H_2_SO_4_. The optical density (OD) was read at 450 nm using a microplate reader (Thermo Fisher Scientific).

#### 2.12.2 Free MMAE and conjugated MMAE analysis by LC‐MS/MS

The concentration of free MMAE and conjugated MMAE in serum was determined by liquid chromatography–tandem mass spectrometry (LC‐MS/MS). The LC‐MS/MS system consisted of Shimadzu LC‐30AD HPLC and API 4000 MS. LC‐MS/MS method: Waters HSS T3 column (1.8 μm, 2.1 × 50 mm); solvent A, 0.1% FA/H_2_O; solvent B, 0.1% FA/acetonitrile; the gradient mode was 10–95% solvent B over 2 min, and 95–10% solvent B over 0.5 min at a flow rate of 0.5 mL·min^−1^; column temperature was 50 °C; transition 718.7/152.2 was monitored for MMAE; the standard curve had a linear range from 0.05 to 200 ng·mL^−1^. For free MMAE analysis, 20 μL samples were precipitated with 60 μL of acetonitrile containing 200 ng·mL^−1^ tolbutamide (internal standard) and then centrifuged to get the supernatant. For conjugate MMAE analysis, 20 μL samples were added 1.6 μL cathepsin B (Sigma‐Aldrich, Saint Louis, MO, USA) and then incubated at 37 °C for 3 h. The reaction was stopped with 60 μL of acetonitrile containing 200 ng·mL^−1^ tolbutamide (internal standard). The conjugated MMAE concentration was calculated using the following formula: (the concentration of hydrolytic MMAE‐the concentration of free MMAE)/hydrolysis rate. The mean time–concentration data were analyzed using WinNonlin to obtain the clearance (CL), maximum concentration (*C*_max_), maximum time (*T*_max_), half‐life (*t*_1/2_), apparent volume of distribution (Vd), mean residence time (MRT), and area under the curve (AUC).

### 2.13 Statistical analysis

The statistical analysis and graphic presentations were performed with origin 8.0 (OriginLab Software, Inc., Northampton, MA, USA) and graphpad prism 5 software (GraphPad Software, Inc., San Diego, CA, USA). The statistical significance of the differences between the two groups was determined using the Student's *t*‐test, and multiple groups were compared using a one‐way analysis of variance (anova) test. *P* values of < 0.05 were accepted as a significant difference. (*P <* *0.05; **P <* *0.01, ***P <* *0.001).

## 3 Results

### 3.1 Characterization of LR004‐VC‐MMAE

The auristatin analogue MMAE is a cytotoxic agent with a pentapeptide structure that inhibits tubulin polymerization. In 2003, Doronina *et al*. reported that MMAE combined with a target‐specific monoclonal antibody increased the therapeutic index (Doronina *et al*., 2003). Generally, MMAE is conjugated with an antibody via an enzymatic degradation linker, called a VC linker. In our experiment, as shown in Fig. S1A–C, MMAE, VC linker (MC‐VC‐PABC‐PNP), and VC‐MMAE (MC‐VC‐PAB‐MMAE) were synthesized according to the published methods (Dubowchik *et al*., 2002; Francisco *et al*., 2003). The purity of the synthesized compound was > 95% by HPLC (Fig. S2A), and the high‐resolution mass spectra (HRMS) were consistent with the structure (Fig. S2B). Notably, the purity of the small molecules ensured the conjugate quality of the ADC.

To validate the characterization of LR004‐VC‐MMAE, the ADC was electrophoretically separated on SDS/PAGE. Under reducing conditions (Fig. 1B), the apparent molecular weight of the light and heavy chains increased slightly compared with the naked antibody. One light chain and one heavy chain carried a maximum of one drug molecule and three drug molecules, respectively. This may be the reason for the slightly elevated molecular weight observed. Under nonreducing conditions (Fig. 1B), LR004 displayed a main band at the apparent molecular weight and another lighter band of a lower molecular weight that likely corresponded to the molecule lacking one light chain. LR004‐VC‐MMAE generated multiple characteristic bands of the distribution of the drug‐linked species. The fractions were distributed as follows: six bands of an apparent molecular weight fit to H2L2, H2L, H2, HL, H, and L (top to bottom), each carrying payloads.

The SEC‐HPLC comparison of the conjugate to the LR004 demonstrated a slight increase in both retention time (LR004,RT = 3.998 s;LR004‐VC‐MMAE, RT = 4.031 s) and peak tailing for the ADC (Fig. 1C). This indicated that the attached hydrophobic drugs led to a nonspecific interaction between the ADC and the column stationary phase. Importantly, SEC‐HPLC showed that the conjugated product was not aggregated. The HIC‐HPLC spectrum demonstrated five major peaks, corresponding to zero, two, four, six, and eight drugs per antibody, and the average drug–antibody ratio (DAR) was approximately 4.0 after the integration of the observed peaks (Fig. 1D). Moreover, the conjugate products in our studies exceeded 97%, and the components of DAR = 4 accounted for more than 40%.

### 3.2 The binding ability of LR004 and LR004‐VC‐MMAE

**Figure 2:**
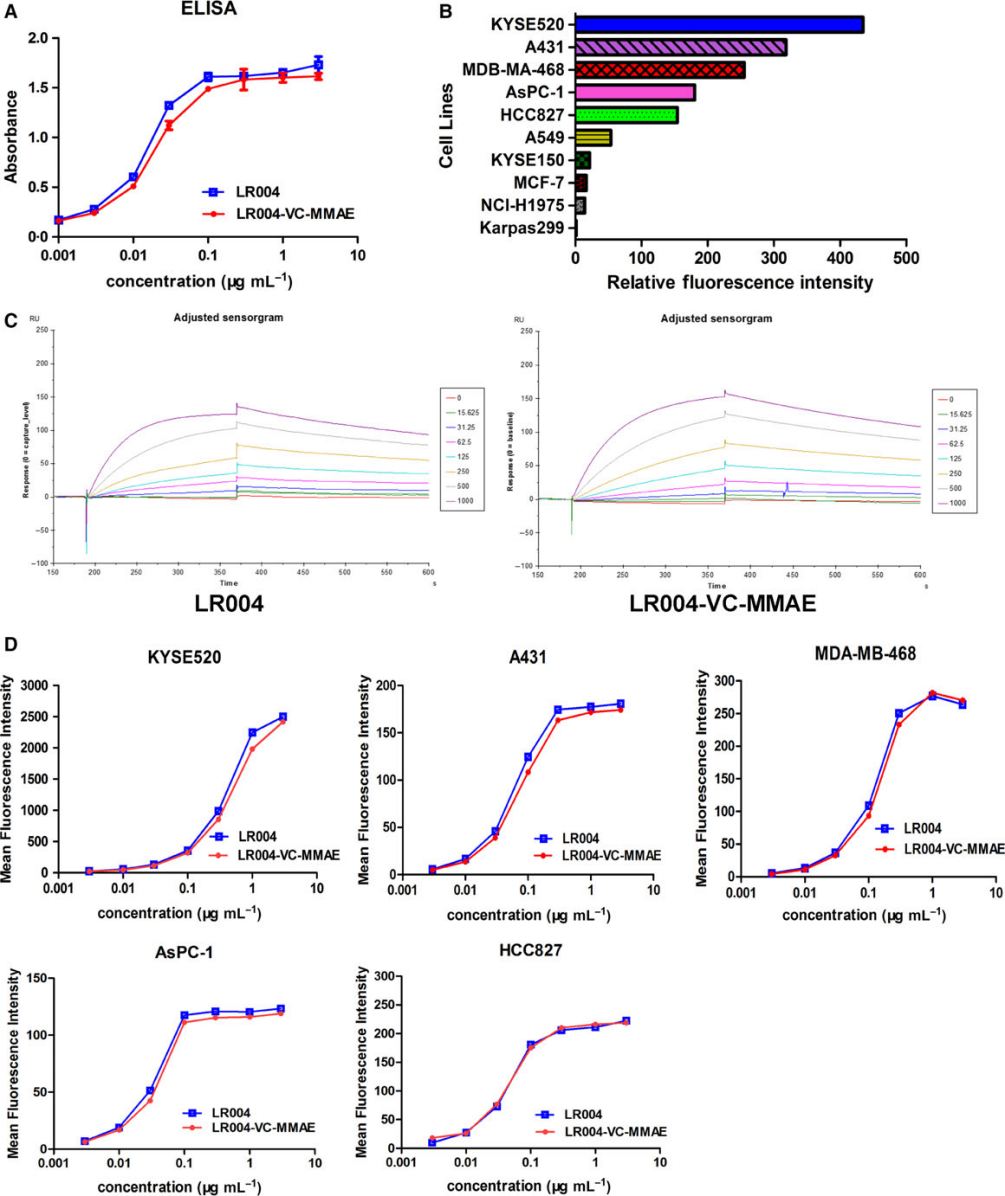
Binding ability of LR004 and LR004‐VC‐MMAE *in vitro*. (A) The binding activity of LR004 and LR004‐VC‐MMAE to the recombinant human EGFR antigen by ELISA. (B) The sensorgram of Biacore analysis. The CM5 sensor chip was pre‐immobilized with LR004 and LR004‐VC‐MMAE at a concentration of 1 μg·mL ^−1^. The antigen EGFR was injected at a flow of 30 μL·min^−1^ at concentrations ranging from 15.625 to 1000 ng·mL ^−1^ in HEPES buffer. The dissociation constant, KD, was calculated as the ratio of these two constants (*k*_off_/*k*_on_). (C) The expression level of EGFR on various cells surface under the saturation state by FACS analysis (the concentration of LR004 was 10 μg·mL ^−1^). The horizontal axis represents the values of relative fluorescence intensity. (D) The binding curves of different concentrations of LR004 and LR004‐VC‐MMAE to the EGFR high‐expression cells by FACS analysis. The vertical axis represents the values of mean fluorescence intensity.

The binding characteristics of LR004 and LR004‐VC‐MMAE were assessed by ELISA, Biacore, and FACS analysis. The binding of LR004 and LR004‐VC‐MMAE to an immobilized EGFR antigen was tested by ELISA. The ELISA‐based binding assay revealed that LR004 and LR004‐VC‐MMAE could bind to the recombinant human EGFR antigen in a concentration‐dependent manner, and the binding ability of LR004‐VC‐MMAE showed a minimal decrease compared to LR004 (Fig. 2A). The binding of LR004 and LR004‐VC‐MMAE to the soluble EGFR antigen was tested by Biacore (Fig. 2C). The *K*D of LR004 was 0.916 nm, which was comparable to LR004‐VC‐MMAE, with a *K*D of 1.313 nm; this suggests that the KD of LR004 and LR004‐VC‐MMAE was high and comparative. The binding of LR004 and LR004‐VC‐MMAE to the cell surface EGFR antigen was tested by FACS. First, the relative fluorescence intensity reflected the expression level of EGFR on the cell surface under the saturation state (LR004 = 10 μg·mL^−1^). As shown in Fig. 2B, the EGFR‐overexpressing cancer cells were the KYSE520, A431, MDA‐MB‐468, AsPC‐1, and HCC827 cells, the EGFR relative low‐expressing cells were the A549, KYSE150, MCF‐7, and NCI‐H1975 cells, and the EGFR‐lacking expression cells were the Karpas 299 cells. Then, the EGFR high‐expression cells were combined with increasing concentrations of LR004 and LR004‐VC‐MMAE, and a result similar to the ELISA was obtained by the FACS‐based binding assay (Fig. 2D), suggesting that the LR004 conjugated with MMAE did not alter the binding ability.

### 3.3 In vitro cytotoxicity of LR004‐VC‐MMAE against ESCC and EGFR‐expressing cells

**Figure 3:**
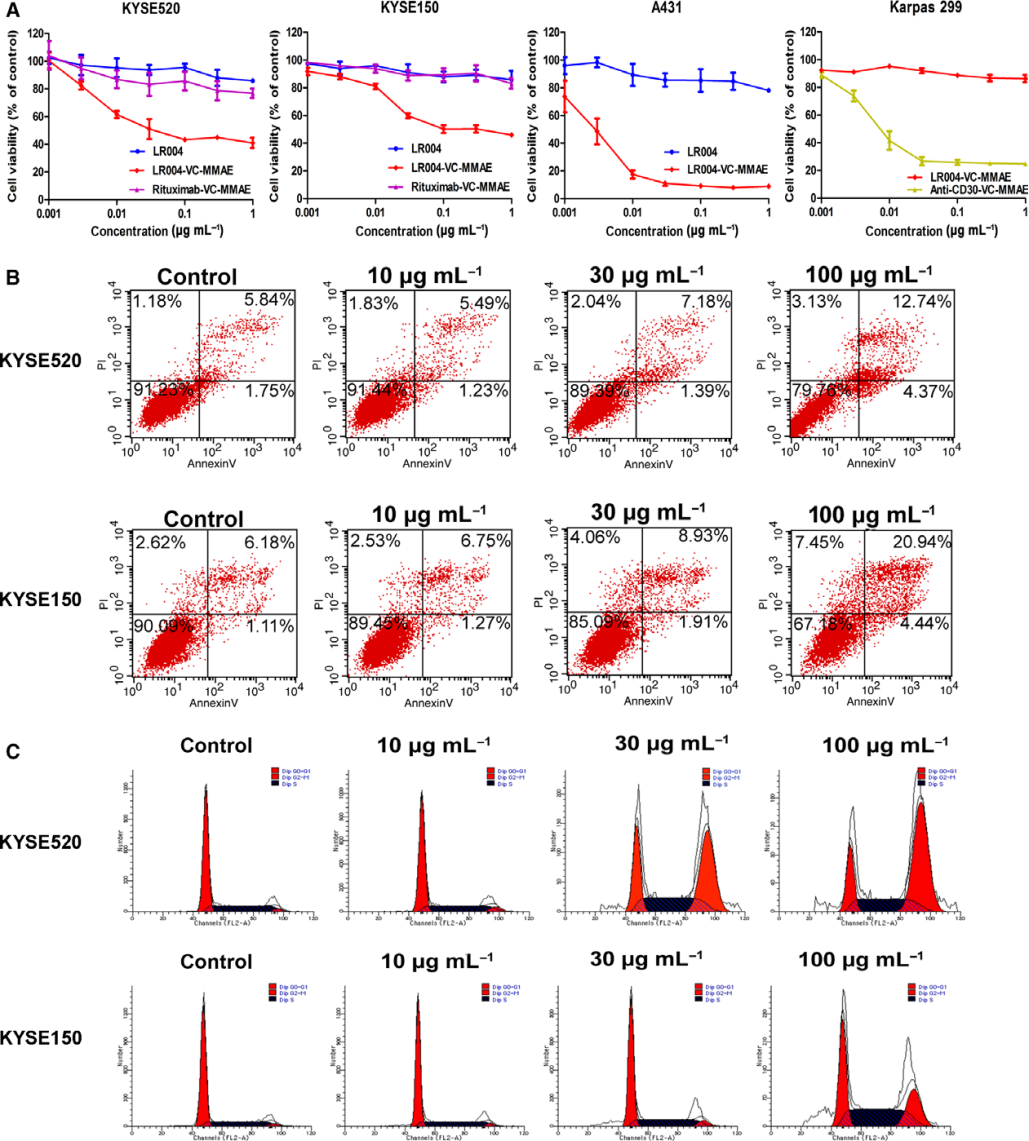
Cytotoxicity *in vitro* of LR004‐VC‐MMAE. (A) The cell viability analysis of LR004 (blue line), LR004‐VC‐MMAE (red line), and rituximab‐VC‐MMAE (purple line) to KYSE520 and KYSE150 cells. The cell viability analysis of LR004‐VC‐MMAE to A431 and Karpas 299 cells. Karpas 299 cells were used as the negative control of cell line which expressed CD30 antigen on the cell surface. The cell viability was assessed using the CCK‐8 assay for 48 h. (B) The induction of apoptosis analysis in the KYSE520 and KYSE150 cells was detected by flow cytometry. The cells were treated with various concentrations of LR004‐VC‐MMAE for 24 h. (C) The cell cycle arrest analysis in the KYSE520 and KYSE150 cells was detected by flow cytometry. The cells were treated with various concentrations of LR004‐VC‐MMAE for 24 h.

LR004‐VC‐MMAE and MMAE displayed significant cytotoxicity against various tumor cells expressing EGFR, with IC_50_ values of 0.01–8 nm (Table 1). In the same type of tumor cells (ESCC, NSCLC, breast cancer), the EGFR‐overexpressing cancer cells were more sensitive to LR004‐VC‐MMAE than the EGFR low‐expressing cells, in terms of the IC_50_ values. In the NSCLC cell lines, LR004‐VC‐MMAE inhibited the viability of the HCC827 and A549 with the IC_50_ of 0.302 ± 0.088 nm and 5.259 ± 1.127 nm, respectively. In the breast cancer cell lines, LR004‐VC‐MMAE inhibited the viability of the MDA‐MB‐468 and MCF‐7 with the IC_50_ of 0.340 ± 0.252 nm and 2.935 ± 0.983 nm, respectively. This suggests that there was a good correlation between EGFR density and the sensitivity to LR004‐VC‐MMAE‐mediated killing. In the ESCC cell lines, the flow cytometry analysis indicated that the KYSE520 cells expressed a high EGFR level (Fig. S4A), while the KYSE150 cells expressed a low relative EGFR level (Fig. S4B). As presented in Fig. 3A, LR004‐VC‐MMAE inhibited the viability of the KYSE520 and KYSE150 cells in a concentration‐dependent manner, and the IC_50_ values were 1.852 ± 0.617 nm and 4.440 ± 0.208 nm, respectively. However, LR004 had a weak inhibitory effect (IC_50_ > 6 nm), implying that the cytotoxicity of LR004‐VC‐MMAE *in vitro* results from the delivery of MMAE rather than from the efficacy of the antibody. To confirm the on‐target killing by LR004‐VC‐MMAE, rituximab‐VC‐MMAE (CD20‐targeting ADC) was used as a negative control, and its minimal cytotoxic activity was observed in the KYSE520 and KYSE150 cells. As presented in Fig. 3A, LR004‐VC‐MMAE inhibited the viability of A431 in a concentration‐dependent manner, with an IC_50_ value of 0.019 ± 0.006 nm, and showed a high tumor growth inhibition ratio (91.17%) at a dose of 1 μg·mL^−1^ (6 nm), whereas the Karpass 299 cells (CD30‐overexpressing cancer cells) were not sensitive to LR004‐VC‐MMAE up to the maximum concentration of 1 μg·mL^−1^ but had superior activity to anti‐CD30‐VC‐MMAE, with an IC_50_ of 0.088 ± 0.002 nm. These results demonstrated that LR004‐VC‐MMAE had selectivity for EGFR‐positive or EGFR‐negative cells.

**Table 1:** IC_50_ values for LR004, LR004‐VC‐MMAE, and MMAE against various EGFR‐expressing cells. The 50% inhibitory concentration (IC_50_) of the samples was calculated by SPSS software. The data are presented as the mean ± SD from three independent experimental titrations

Tumor types	Cell line	IC_50_ (nm)		
		LR004	LR004‐VC‐MMAE	MMAE
ESCC	KYSE520	> 6	1.852 ± 0.617	0.490 ± 0.113
	KYSE150	> 6	4.440 ± 0.208	1.835 ± 0.160
Epidermoid carcinoma	A431	> 6	0.019 ± 0.006	0.016 ± 0.013
NSCLC	HCC827	> 6	0.302 ± 0.088	1.952 ± 0.312
	NCI‐H1975	> 6	0.932 ± 0.227	0.036 ± 0.013
	A549	> 6	5.259 ± 1.127	0.316 ± 0.078
Breast cancer	MDA‐MB‐468	> 6	0.340 ± 0.252	3.279 ± 0.950
	MCF‐7	> 6	2.935 ± 0.983	0.714 ± 0.089
Pancreatic cancer	AsPC‐1	> 6	0.176 ± 0.069	0.648 ± 0.221
ALCL	Karpas 299	> 60	> 60	0.218 ± 0.154

To assess the induction of apoptosis and cell cycle, the ESCC cells and A431 cells were incubated with various concentrations of LR004‐VC‐MMAE for 24 h. The flow cytometry analysis indicated that the ratio of apoptosis and dead cells in the tested ESCC and A431 cells increased in a concentration‐dependent manner (Figs 3B and S5A). The flow cytometry analysis clearly showed a significant loss of G1 DNA content and an increase in G2 DNA content in a concentration‐dependent manner (Figs 3C and S5B). These data suggest that MMAE appended to LR004 can induce cell apoptosis and produce a potent G2/M arrest in ESCC cells and A431 cells.

### 3.4 EGFR‐mediated endocytosis and intracellular trafficking

**Figure 4:**
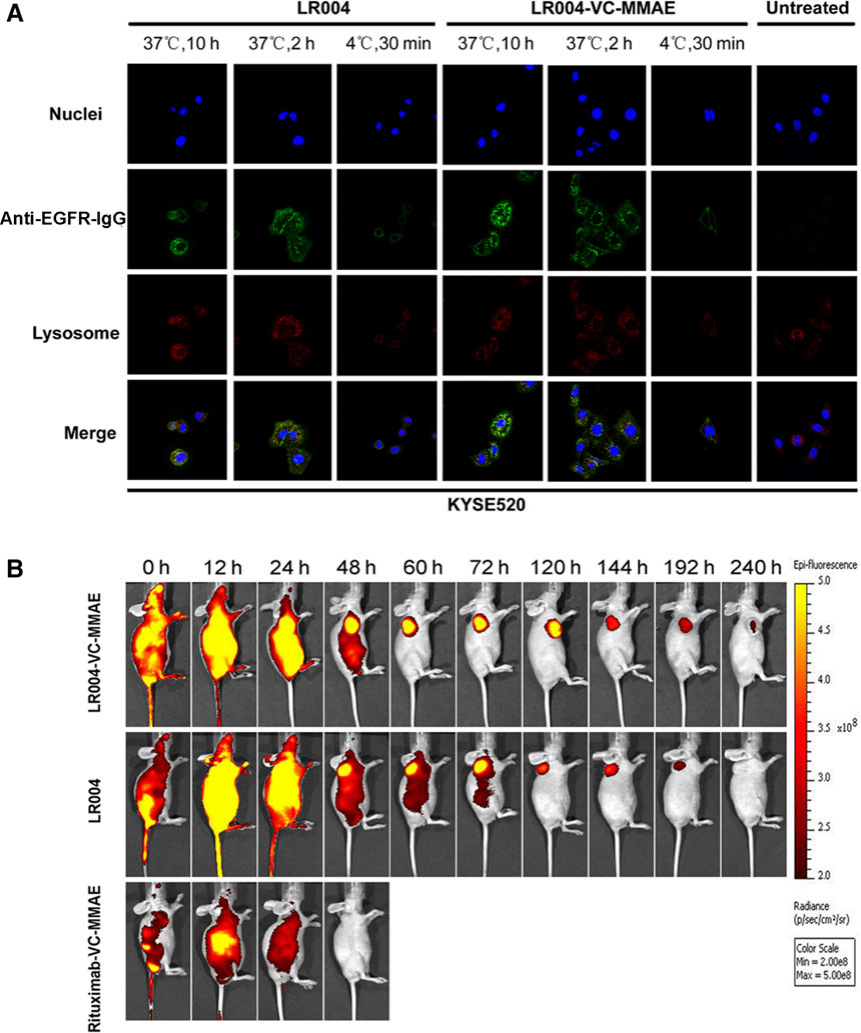
Confocal analysis for intracellular localization and fluorescence imaging in KYSE520 model. (A) The internalization and lysosomal localization of LR004 and LR004‐VC‐MMAE in the KYSE520 cells by laser scanning confocal microscope. The KYSE520 cells were treated with 5 μg·mL ^−1^ LR004 and LR004‐VC‐MMAE at 4 °C for 30 min or at 37 °C for 2 and 10 h. The lysosomes were labeled with a LAMP‐1 antibody followed by an Alexa Fluor 555‐labeled goat anti‐rabbit IgG (H+L) antibody. The cell nuclei were stained with DAPI. (B) *In vivo* fluorescence imaging of LR004 and LR004‐VC‐MMAE in KYSE520 nude mice xenograft model. Mice in the three DyLight 680‐labeled groups (LR004, LR004‐VC‐MMAE, and rituximab groups) were injected via the tail veins with the dose of 20 mg·kg^−1^ each. Representative *in vivo* fluorescence imaging at the indicated time points. Color scale represents photons/s/cm^2^/steradian.

The internalization and lysosomal localization of LR004 and LR004‐VC‐MMAE were detected in the KYSE520 cells by laser scanning confocal microscope. LR004 and LR004‐VC‐MMAE were bound to the cell peripheral membrane of KYSE520 cells at 4 °C for 30 min, while the anti‐EGFR‐IgG staining (green) was not localized inside the cells or colocalized with the lysosomal markers. Upon elevating the temperature to 37 °C for 0.5 h, the anti‐EGFR‐IgG staining shifted to a capping and punctate pattern (Fig. 4A), with LR004 and LR004‐VC‐MMAE colocalized within Lamp‐1. At 10 h, internalized LR004 and LR004‐VC‐MMAE was still present in the lysosomes, indicating that both LR004 and LR004‐VC‐MMAE were rapidly internalized into the cells and continuously transported to the lysosomes in KYSE520 cells.

### 3.5 In vivo fluorescence imaging of LR004 and LR004‐VC‐MMAE

The *in vivo* tissue distribution and targeting accumulation capability of LR004, LR004‐VC‐MMAE, and rituximab‐VC‐MMAE were evaluated in a nude mice KYSE520 xenograft model via an optical molecular imaging system. After IV administration to the mice in the three Dylight 680‐labeled groups at a dosage of 20 mg·kg^−1^ each, LR004 and LR004‐VC‐MMAE showed a higher fluorescence than rituximab‐VC‐MMAE within 24 h. Then, the fluorescence signal of LR004 and LR004‐VC‐MMAE was initially visualized in the tumor sites within 48 h, and the tumor‐located image was clearly maintained for 6 days (Fig. 4B). In contrast, the negative control rituximab‐VC‐MMAE did not demonstrate targeting accumulation in the tumor sites and was metabolized by the liver and kidney at 24 h postinjection. As described above, there was strong selective tumor accumulation and localization in the EGFR‐positive KYSE520 tumors with LR004 and LR004‐VC‐MMAE.

### 3.6 Efficacy of LR004‐VC‐MMAE in nude mice bearing ESCC xenografts

**Figure 5:**
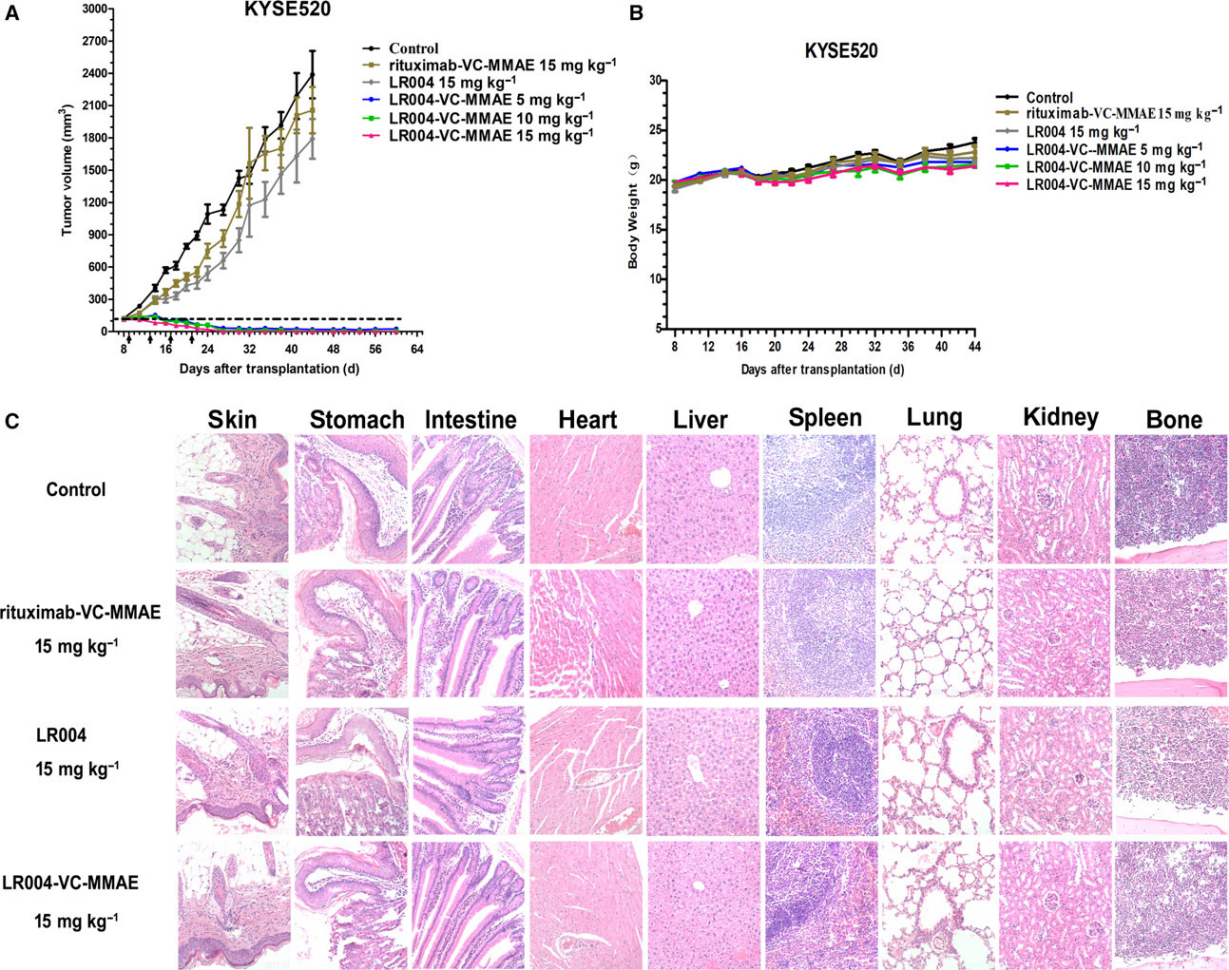
Therapeutic efficacy of LR004‐VC‐MMAE against KYSE520 tumor xenograft model in nude mice. (A) Tumor growing curves of ESCC KYSE520 tumor xenograft model (*n* = 6). The mice were treated with various doses of LR004‐VC‐MMAE (5, 10, 15 mg·kg^−1^), LR004 (15 mg·kg^−1^), and rituximab‐VC‐MMAE (15 mg·kg^−1^) every 4 days for a total of four injections. (B) Body weight change of the KYSE520 tumor xenograft model. (C) Histopathological examination (H&E staining, ×200) of various organs and tumors (skin, heart, liver, spleen, lung, kidney, stomach, intestine, and bone) of the KYSE520 xenograft tumor nude model treated with the medicated groups.

The EGFR‐overexpressing ESCC KYSE520 xenograft model was designed to assess the ability of LR004‐VC‐MMAE to mediate antitumor activity. Tumors in animals treated with the negative control rituximab‐VC‐MMAE (15 mg·kg^−1^) grew from an average of 123 ± 18 mm^3^ on day 8 to an average of 2251 ± 520 mm^3^ on day 44 after implantation. The slight growth inhibition of rituximab‐VC‐MMAE may be a result of antibody or ADC accumulation (Boghaert *et al*., 2006; Sano *et al*., 2013). We have shown that repeated dosing with the naked antibody LR004 at 15 mg·kg^−1^ did not induce regression (average tumor volume 1791 ± 489 mm^3^, tumor inhibition rate was 13.8% on day 44; *P *=* *0.3018), suggesting that the LR004 antibody, as a single agent, had minimal activity in this model. Tumor regression was significantly observed in all the animals treated with 5 mg·kg^−1^ of LR004‐VC‐MMAE (0.1 mg MMAE·kg^−1^; *P *<* *0.0001), 10 mg·kg^−1^ of LR004‐VC‐MMAE (0.2 mg MMAE·kg^−1^; *P *<* *0.0001), and 15 mg·kg^−1^ of LR004‐VC‐MMAE (0.3 mg MMAE·kg^−1^; *P *<* *0.0001) compared with both the control and LR004 (Fig. 5A). The tumors disappeared in 6 mice (6/8) in the group receiving 5 mg·kg^−1^ on day 44 and disappeared in 7 (7/8) mice in the group receiving 10 mg·kg^−1^ on day 41. At the 15 mg·kg^−1^ dose level, 8 animals (8/8) achieved complete regression on day 27. Surprisingly, tumor recurrence was not observed until the experiment was terminated on day 60. None of the treatments produced death or body weight loss in the mice (Fig. 5B). In addition, we assessed the toxicity of LR004 and LR004‐VC‐MMAE at a dosage of 15 mg·kg^−1^. There were no apparent abnormalities in the skin of the mice, and no toxico‐pathological changes were found in other organs (Fig. 5C). These results confirmed that LR004 and its ADC has a low systemic toxicity in the mice model.

### 3.7 Efficacy of LR004‐VC‐MMAE in nude mice bearing A431 xenografts

**Figure 6:**
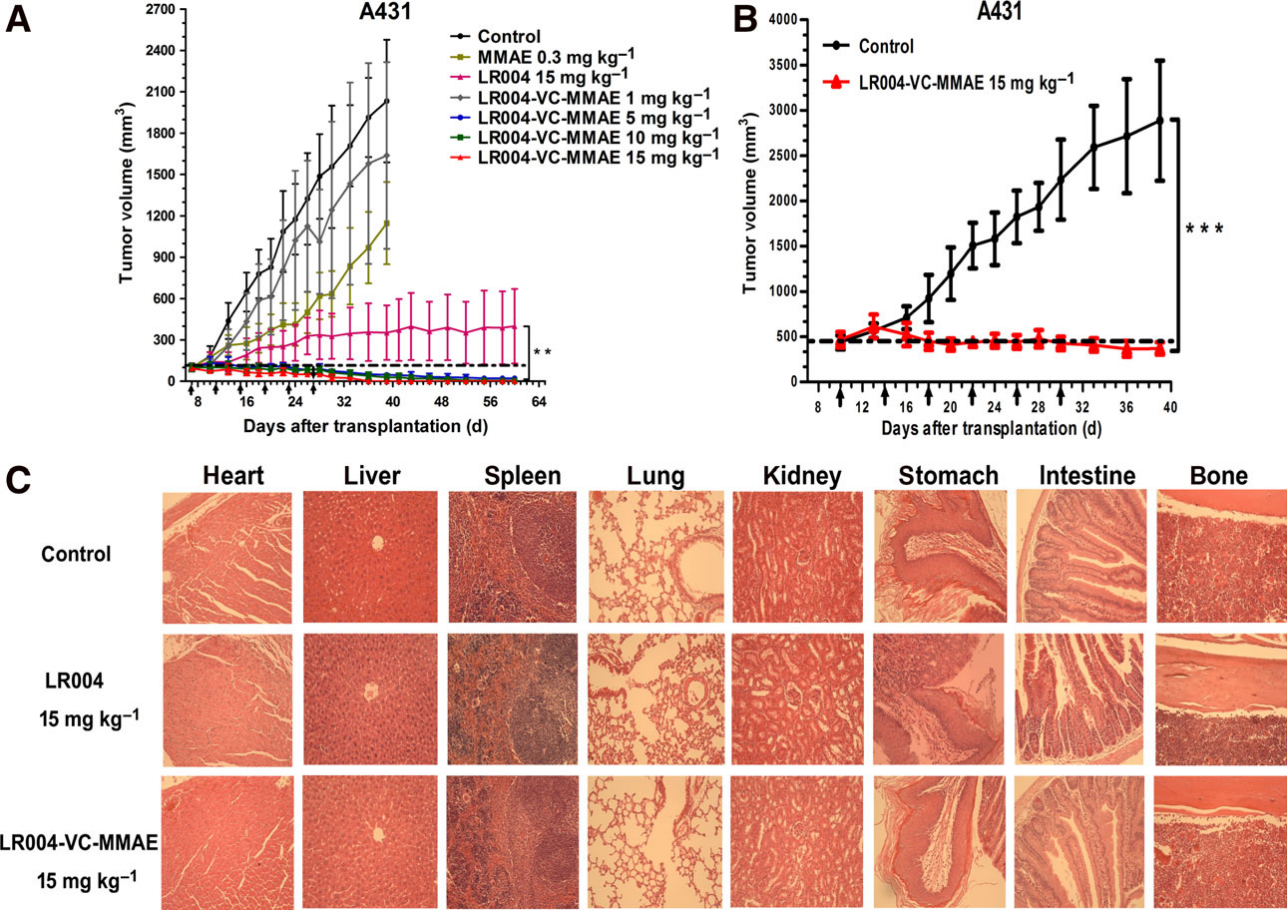
Therapeutic efficacy of LR004‐VC‐MMAE against A431 tumor xenograft model in nude mice. (A) Tumor growing curves of the A431 tumor xenograft model (*n* = 7). The mice were treated with various doses of LR004‐VC‐MMAE (1, 5, 10, 15 mg·kg^−1^), LR004 (15 mg·kg^−1^) and MMAE (0.3 mg·kg^−1^) every 4 days for a total of six injections. ***P <* *0.0001, compared with the control and MMAE (0.3 mg·kg^−1^) groups on day 40. **P <* *0.01, compared with the LR004 (15 mg·kg^−1^) on day 60. (B) Tumor growing curves for the large tumor group of the A431 tumor xenograft model. ***P <* *0.0001, compared with the control. (C) Histopathological examination (H&E staining, ×200) of various organs and tumors (heart, liver, spleen, lung, kidney, stomach, intestine, and bone) of the A431 xenograft tumor nude model treated with LR004 and LR004‐VC‐MMAE at a dosage of 15 mg·kg^−1^, respectively.

In the A431 xenograft model, animals bearing the A431 xenograft were randomized for treatment in seven groups when the tumor volume reached approximately 100 mm^3^ (Fig. 6A). LR004‐VC‐MMAE significantly delayed tumor growth at the 5, 10, and 15 mg·kg^−1^ dose levels compared with the control group on day 40 (*P *<* *0.0001). From days 40 to 60, all the animals treated with LR004‐VC‐MMAE in the three dose groups possessed tumors less than 33 mm^3^ in size and even appeared tumor complete regressions. However, free MMAE (0.3 mg·kg^−1^, a dose equivalent to the dose administered as the LR004‐VC‐MMAE 15 mg·kg^−1^) presented little inhibitory effect (average tumor volume 1147 ± 272 mm^3^, tumor inhibition rate was 43.6% on day 40). In contrast, LR004 (15 mg·kg^−1^), as a single agent, suppressed tumor growth in this model on day 60 but had a limited therapeutic effect compared with LR004‐VC‐MMAE (average tumor volume 399 ± 246 mm^3^; *P *<* *0.01 compared with the three ADC groups), indicating that the conjugation of MMAE significantly increased potency. By H&E, no toxico‐pathological changes were found in various organs after treatment with LR004 and LR004‐VC‐MMAE at the maximum tolerated dosage of 15 mg·kg^−1^ after six injections (Fig. 6C).

For the second study, mice were treated with either a dose of 15 mg·kg^−1^ of LR004‐VC‐MMAE or a control when the tumor volume reached a size of 400–500 mm^3^. The control group showed progressive tumor growth, with an average TV of more than 2500 mm^3^ within 40 days, whereas LR004‐VC‐MMAE suppressed tumor growth or even reduced tumor size, and the tumor inhibition rate increased to 87.2% (average tumor volume 370 ± 62 mm^3^; *P *<* *0.0001). (Fig. 6B).

### 3.8 Pharmacokinetic studies of LR004‐VC‐MMAE in nude mice model

**Figure 7:**
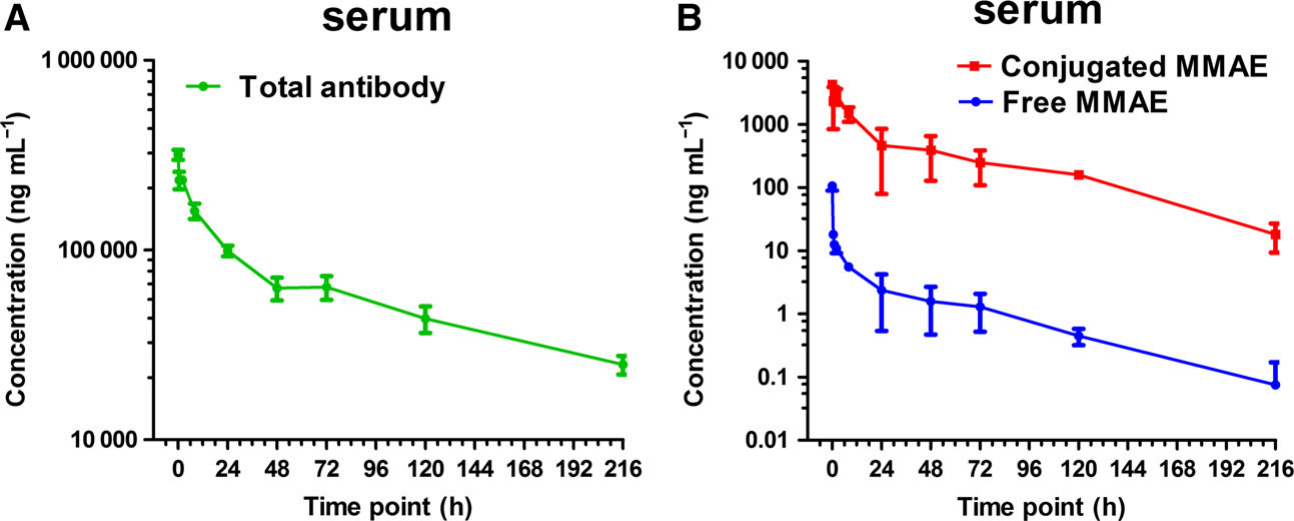
Concentration–time curves of single dose administrated with LR004‐VC‐MMAE in nude mice model. The BALB/c nude mice were injected subcutaneously with 15 mg·kg^−1^ of LR004‐VC‐MMAE and then sacrificed at 0, 0.5, 1, 2, 6, 24, 48, 72, 120, and 216 h after serum collection. (A) The mean total antibody concentrations (by ELISA) in serum of LR004‐VC‐MMAE ADC in nude mice. (B) The mean free MMAE (blue curve) and conjugated MMAE (red curve) concentrations (by LC‐MS/MS) in serum following administration of LR004‐VC‐MMAE in nude mice. Data points represent mean ± SD,* n* = 4 in both (A) and (B).

The BALB/c nude mice were injected subcutaneously with 15 mg·kg^−1^ of LR004‐VC‐MMAE, and then, both sera were sampled at 0, 0.5, 1, 2, 6, 24, 48, 72, 120, and 216 h. Changes in total antibody concentration are driven solely by elimination of ADC and unconjugated antibody. The concentration of total antibodies (antibodies with and without MMAE attached) varied with time in serum as shown in Fig. 7A. The PK profile of total antibody of LR004‐VC‐MMAE had the elimination half‐life (t_1/2_) of 113.61 ± 20.07 h (~ 5 days) and the clearance (CL) at rates of 0.88 ± 0.05 mL·h^−1^·kg^−1^ *in vivo*. The calculated total antibody volume of distribution (Vd) was 0.14 ± 0.03 L·kg^−1^ at 15 mg·kg^−1^ dose. Serum concentration–time profiles of free MMAE and conjugated MMAE, measured by LC‐MS/MS, were presented in Fig. 7B. Observed free MMAE concentrations were generally very low, the pharmacokinetics data revealed that concentrations of free MMAE declined to 2.4 ± 1.84 ng·mL^−1^ over 24 h. However, LR004‐VC‐MMAE showed a higher concentration of conjugated MMAE at time points ranging from 0.5 h to 5 days. Changes in conjugated drug concentration could reflect both elimination of ADC from systemic circulation and loss of cytotoxic drug from the antibody. The PK profile of conjugated MMAE of LR004‐VC‐MMAE had the elimination half‐life (t_1/2_) of 33.31 ± 6.15 h and the clearance (CL) at rates of 3.67 ± 0.50 L·h^−1^·kg^−1^ *in vivo*. The Cmax and the AUC_0‐∞_ value of conjugated MMAE were 4288.8 ± 426.5 ng·mL^−1^ and 71 929.1 ± 10 470.1 h·ng·mL^−1^, respectively. A summary of the PK parameters for LR004‐VC‐MMAE total antibody, conjugated MMAE, and free MMAE in serum was presented in Tables S1, S2 and S3.

## 4 Discussion

Exploiting molecular targets such as an EGFR‐targeting ADC is one strategy for developing therapeutics with the potential to improve antitumor activity in some solid malignancies. LR004 is of great value as a carrier in ADCs with high binding affinity and internalization ability. These attributes were supported by the results presented here. First, LR004 was bound to various forms of the EGFR antigen with a high affinity. A high binding affinity (KD < 1 nm) ensures good tumor localization and decreases nonselective toxicity toward normal cells (Adams *et al*., 2001). Second, LR004 was rapidly internalized into the cell followed by localization in the lysosomes. This internalization process, called receptor‐mediated endocytosis, is an important factor for the antibody of the ADC. Therefore, we hypothesized that LR004 could directly delivery the highly potent antitubulin drug MMAE to display a potential antitumor activity. MMAE may diffuse from the target cells and subsequently permeate adjacent cells that need not be positive for the target antigen (Katz *et al*., 2011). This bystander cytotoxicity may be beneficial when treating solid tumors that are either homogeneous or heterogeneous (Kovtun *et al*., 2006; Li *et al*., 2016). In our study, MMAE, linker VC, and VC‐MMAE were synthesized by our laboratory with high purity. LR004 was covalently conjugated with MMAE via cysteine residues released by an antibody interchain disulfide bond reduction. LR004‐VC‐MMAE had a theoretical DAR distribution of 0, 2, 4, 6, and 8 isoforms (average DAR is approximately 4.0), and the coupling technology was shown to be stable and controllable. Moreover, it was reported that a DAR of 4, through cysteine residue conjugation, yields the best therapeutic effect (Hamblett *et al*., 2004). In this study, the components of four drugs/antibody accounted for more than 40%, and the nonconjugated antibody was lower than 3%.

Esophageal squamous cell carcinoma represents a significant global health problem, especially in China (Chen *et al*., 2016). There is an urgent need to develop a more effective agent to control this disease. ADC may possibly offer the promise of an increased therapeutic index for ESCC. In this study, we evaluated the functional characterization and antitumor activities of LR004‐VC‐MMAE on ESCC cell lines *in vitro* and *in vivo*. It is well known that EGFR was purified initially from A431 cells, which overexpress EGFR from 2‐ to 100‐fold (Wrann and Fox, 1979). We further explored whether LR004‐conjugated MMAE could significantly improve the antitumor activity in A431 model. *In vitro*, LR004‐VC‐MMAE showed a promising cytotoxicity on ESCC cells, while the cytotoxicity of LR004 was nearly undetectable. Notably, the A431 cell viability was reduced significantly when it was incubated with the LR004‐VC‐MMAE. The result from the cell cycle arrest and apoptosis analysis to ESCC cells and A431 cells demonstrated that the LR004‐VC‐MMAE caused G2/M arrest and induced significant apoptosis. This mechanism was specifically induced by the MMAE delivered by the ADC. Furthermore, it is reported that the delivery of molecular payloads by antibodies is limited by the efficient internalization of the antigen–antibody conjugate complex (Chari *et al*., 2014). The LR004‐VC‐MMAE uptake in the KYSE520 cells was visible within 0.5 h with a significant surface signal remaining after 10 h, thus indicating that the cytotoxin could be released to kill the cells rapidly and continuously.

The antitumor activity of LR004‐VC‐MMAE in the ESCC KYSE520 tumor model was evaluated in these experiments. LR004‐VC‐MMAE effectively eliminated the tumors and prevented recurrence. Significant activity was seen at 5 mg·kg^−1^, and complete tumor regression was observed at 15 mg·kg^−1^ in all the mice after four injections. However, LR004 showed little effect on the inhibition of tumor growth at the 15 mg·kg^−1^ dose level. Interestingly, both LR004 and LR004‐VC‐MMAE presented tumor accumulation and localization by the *in vivo* fluorescence imaging experiment. For the anti‐EGFR antibody, it is reported that the antibody, as a single agent, has minimal clinical activity in patients with ESCC (Chan *et al*., 2011). The main reason for this may be that the key mechanism of the EGFR antibody is achieved by blocking the EGFR signaling pathway when accumulating around the tumor, whereas gene mutations and amplifications of the EGFR downstream signaling pathways are frequently noted in ESCC (Song *et al*., 2014; Zhou *et al*., 2017). It suggests that the low response rate to LR004 in the ESCC KYSE520 tumor model might be due to high frequency of gene alteration of EGFR downstream signaling pathways. Recent studies have pointed to the potential for combinations of anti‐EGFR antibody with TKIs to overcome the resistance associated with EGFR mutations (Cavazzoni *et al*., 2012). However, such strategies may be limited by the overlapping toxicities. For the ADC, it is important that ADC is mainly considered to depend on cell membranous EGFR expression, but not on the intracellular EGFR signaling cascades. The antibody, as a carrier, targets to the tumors effectively, and then, the tumor‐targeting capability exerts an important role for delivery of cytotoxic payloads to cancer cells. The results we observed suggest that the LR004‐conjugated MMAE significantly increases the antitumor activity of the parental antibody LR004 to treat ESCC. Therefore, our data suggest that the LR004‐conjugated MMAE may provide promising support for new agent development against ESCC.

We extended our studies into xenograft models using A431 cells, and similar promising results were obtained. The activity of LR004‐VC‐MMAE was still significant compared with MMAE. Although the parental antibody LR004 also exhibited attractive activity, as reported, the data were statistically significant compared to LR004‐VC‐MMAE. In the large TV (400–500 mm^3^) group for the A431 models, LR004‐VC‐MMAE, at 15 mg·kg^−1^, led to a significant inhibition of tumor volume, demonstrating that the tumors remained responsive to LR004‐VC‐MMAE in the large tumor experiments.

Furthermore, we showed that EGFR was differentially expressed in other cells lines such as NSCLC cells, breast cancer, and pancreatic cancer cells. The FACS binding analyses of the cells highly expressing EGFR confirmed the kinetic binding affinity of LR004‐VC‐MMAE for the cell surface receptor, with binding characteristics that were essentially indistinguishable from LR004. All the tumor model cells expressing EGFR were assessed *in vitro*, and the results showed potent cytotoxicity to LR004‐VC‐MMAE, with IC_50_ values of nm concentrations. We found that the difference in the sensitivity to the same type of tumor cell may be correlated with the EGFR expression level, but this is not the only crucial factor. Other factors may also contribute to the activity, such as cell cycle, the rate of internalization, and the sensibility of MMAE. In addition, the activity of LR004‐VC‐MMAE and MMAE was comparative, but the ADC showed poor activity in the EGFR‐negative cell Karpas 299. Because Karpas 299 cells lack EGFR expression and the ADC could not specifically bind to the target antigen and undergo internalization to release MMAE. In other words, this targeted delivery improves the selectivity of cytotoxic agents as the warhead. Therefore, our study suggested that LR004‐VC‐MMAE might be one such promising treatment for EGFR‐positive malignancies.

PK profiles of LR004‐VC‐MMAE total antibody and MMAE (free MMAE and conjugated MMAE) were measured by ELISA and LC‐MS/MS, respectively. In mouse experimental model, LR004‐VC‐MMAE total antibody presented a longer half‐life and a lower clearance. The PK parameters for total antibody are in the range typical for an antibody. The result indicated that LR004 with MMAE has not compromised the characteristics of the antibody *in vivo* and supported the LR004 as a suitable delivery vehicle for an ADC carrier. When comparing total antibody PK with conjugated MMAE PK (Fig. S6), it was observed that conjugated MMAE concentrations started at higher concentration than total antibody, reflecting its DAR (DAR_˜4_ to DAR~_0_ transition), the intersection point of two concentration–time curves reflected that the average DAR equaled one. Finally, the conjugated MMAE concentrations decreased more rapidly than total antibody due to antibody elimination and cytotoxic drug deconjugation (Lin and Tibbitts, 2012). Free MMAE released from the ADC is a concern and may be associated with loss of efficacy or increased toxicity. Very low levels of free MMAE were detected for LR004‐VC‐MMAE ADC in systemic circulation. This reflected the stability of the linker and relatively limited deconjugation of MMAE from conjugated LR004, thus avoiding inducing severe systemic toxicity. According to the result of intracellular trafficking and *in vivo* fluorescence imaging analysis, on the other hand, it indicated that LR004‐VC‐MMAE could effectively release MMAE in target tumor cells. Overall, the preliminary pharmacokinetic evaluation can provide some guidance for LR004‐VC‐MMAE ADC in malignancies.

## 5 Conclusion

In conclusion, the ADC of LR004‐VC‐MMAE could be a potential therapeutic agent for ESCC and other EGFR‐expressing malignancies, with a combination of antitumor activity and a desirable PK and safety profile in the mice model. The therapeutic efficacy and safety of the LR004‐based ADC deserve further investigation in other relevant toxicology species.

## Conflict of interest

The authors declare no conflict of interest.

## Authors contributions

ZL and QM designed this study. XH and RW contributed to the collection, analysis, and interpretation of the data. XH drafted the manuscript. XL provided support in the animal studies. JJ helped in preparation of the ADC. AC, LS, and YW helped in generating and purifying the small molecule. YL performed the H&E staining analysis. ZL, QM, and YZ reviewed the manuscript.

## Supporting information


**Fig. S1.** Structure and synthetic route of MMAE, VC and VC‐MMAE.
**Fig. S2.** Characterization of MMAE, VC and VC‐MMAE.
**Fig. S3.** HIC analysis of rituximab‐VC‐MMAE and anti‐CD30‐VC‐MMAE.
**Fig. S4.** The expression level of EGFR on the ESCC cell lines surface by FACS analysis.
**Fig. S5.** Apoptosis and cell cycle arrest analysis in A431 cells by flow cytometry.
**Fig. S6.** PK profile of total antibody, conjugated MMAE and free MMAE administrated with LR004‐VC‐MMAE in nude mice model.
**Table S1.** Pharmacokinetic parameters of total antibody in nude mice.
**Table S2.** Pharmacokinetic parameters of conjugated MMAE in nude mice.
**Table S3.** Pharmacokinetic parameters of free MMAE in nude mice.
