# Supplementary material for: An EGFR‐targeting antibody–drug conjugate LR004‐VC‐MMAE: potential in esophageal squamous cell carcinoma and other malignancies
Source: Mol Oncol. 2018 Nov 15;13(2):246–63. doi: 10.1002/1878-0261.12400 (PMC6360372; doi:10.1002/1878-0261.12400)
Supplement: Supplementary file 1 — Fig. S1. Structure and synthetic route of MMAE, VC and VC‐MMAE. Fig. S2. Characterization of MMAE, VC and VC‐MMAE. Fig. S3. HIC analysis of rituximab‐VC‐MMAE and anti‐CD30‐VC‐MMAE. Fig. S4. The expression level of EGFR on the ESCC cell lines surface by FACS analysis. Fig. S5. Apoptosis and cell cycle arrest analysis in A431 cells by flow cytometry. Fig. S6. PK profile of total antibody, conjugated MMAE and free MMAE administrated with LR004‐VC‐MMAE in nude mice model. Table S1. Pharmacokinetic parameters of total antibody in nude mice. Table S2. Pharmacokinetic parameters of conjugated MMAE in nude mice. Table S3. Pharmacokinetic parameters of free MMAE in nude mice. [file MOL2-13-246-s001.docx]

**Additional Supporting Information:**

**Figure S1. Structure and synthetic route of MMAE, VC and VC-MMAE.**

**Figure S2. Characterization of MMAE, VC and VC-MMAE.**

**Figure S3. HIC analysis of rituximab-VC-MMAE and anti-CD30-VC-MMAE.**

**Figure S4. The expression level of EGFR on the ESCC cell lines surface by FACS analysis.**

**Figure S5. Apoptosis and cell cycle arrest analysis in A431 cells by flow cytometry.**

**Figure S6. PK profile of total antibody, conjugated MMAE and free MMAE administrated with LR004-VC-MMAE in nude mice model.**

**Table S1. Pharmacokinetic parameters of total antibody in nude mice.**

**Table S2. Pharmacokinetic parameters of conjugated MMAE in nude mice.**

**Table S3. Pharmacokinetic parameters of free MMAE in nude mice**

**

**

**Figure S1.** **Structure and synthetic route of MMAE, VC and VC-MMAE. (**a) The structure and synthetic route of MMAE. (b) The structure and synthetic route of VC. (c) The structure and synthetic route of VC-MMAE.


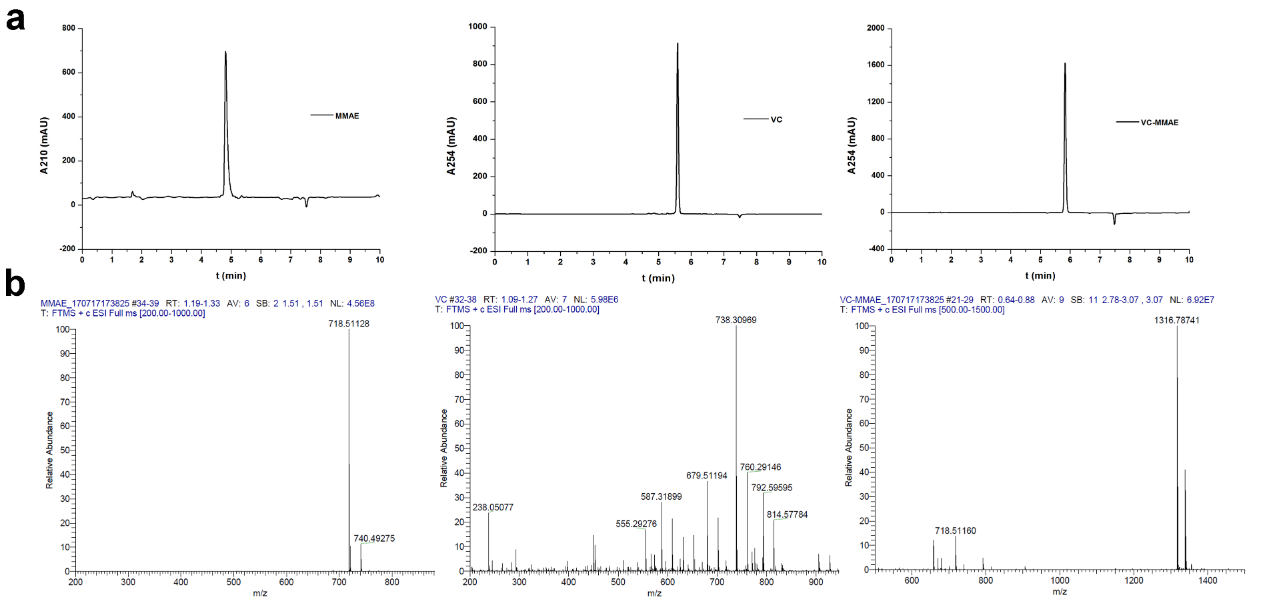


**Figure S2.** **Characterization of MMAE, VC and VC-MMAE.** (a) HPLC analysis of MMAE, VC and VC-MMAE. HPLC method: 1200 Series Instrument (Agilent Technologies); Eclipse XDB-C18 (4.6×150 mm, 5 µm); Solvent A: 0.08% TFA/H_2_O; Solvent B: 0.08% TFA/acetonitrile. The gradient mode was 10%-90% solvent B over 5 min, and 90%-10% solvent B over 5 min at a flow rate of 1 mL/min. VC and VC-MMAE were dectected at 254 nm and MMAE was dectected at 210 nm. (b) HRMS analysis of MMAE, VC and VC-MMAE. HRMS was taken on a LTQ Orbitrap XL instrument (Thermo Scientific, USA). All purified compounds were analyzed using a Bruker 500 MHz spectrometer (Bruker Bioscience, USA).


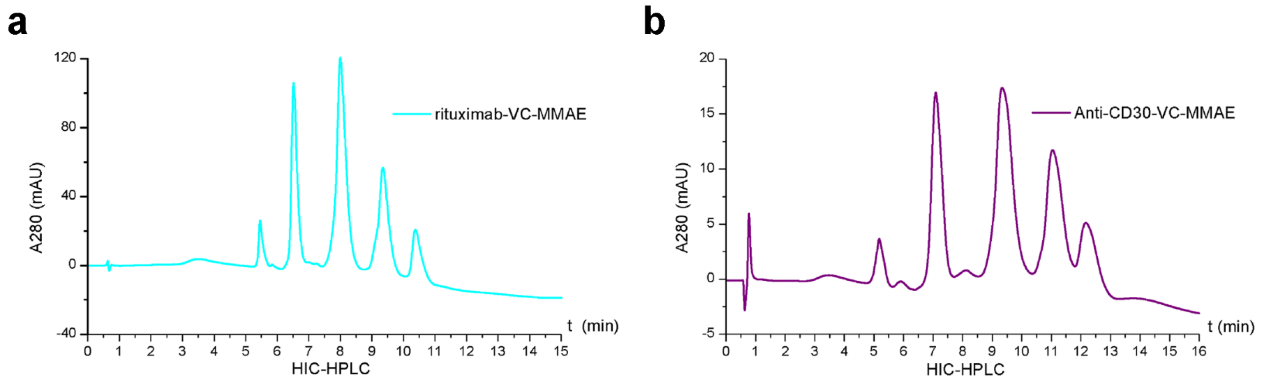


**Figure S3.** **HIC analysis of** **rituximab-VC-MMAE and anti-CD30-VC-MMAE.** (a) HIC analysis of rituximab-VC-MMAE. The average DAR is 3.9 (approximately 4.0) after integration of the observed peaks. (b) HIC analysis of anti-CD30-VC-MMAE. The average DAR is 4.3 (approximately 4.0) after integration of the observed peaks.


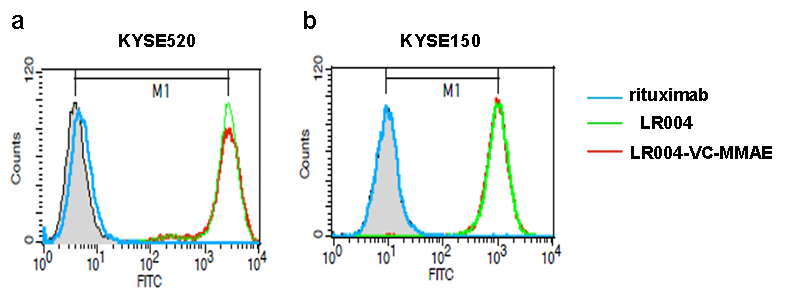


**Figure S4.** **The expression level of EGFR on the ESCC cell lines surface by FACS analysis.** (a) The expression level of EGFR on KYSE520 cells surface. (b) The expression level of EGFR on KYSE150 cells surface. Cells were treated with LR004 and LR004-VC-MMAE at the concentration of 10 μg/mL, respectively. The horizontal axis represents the values of mean fluorescence intensity. The binding of rituximab (10 μg/mL) to ESCC cell lines were used as a negative control.


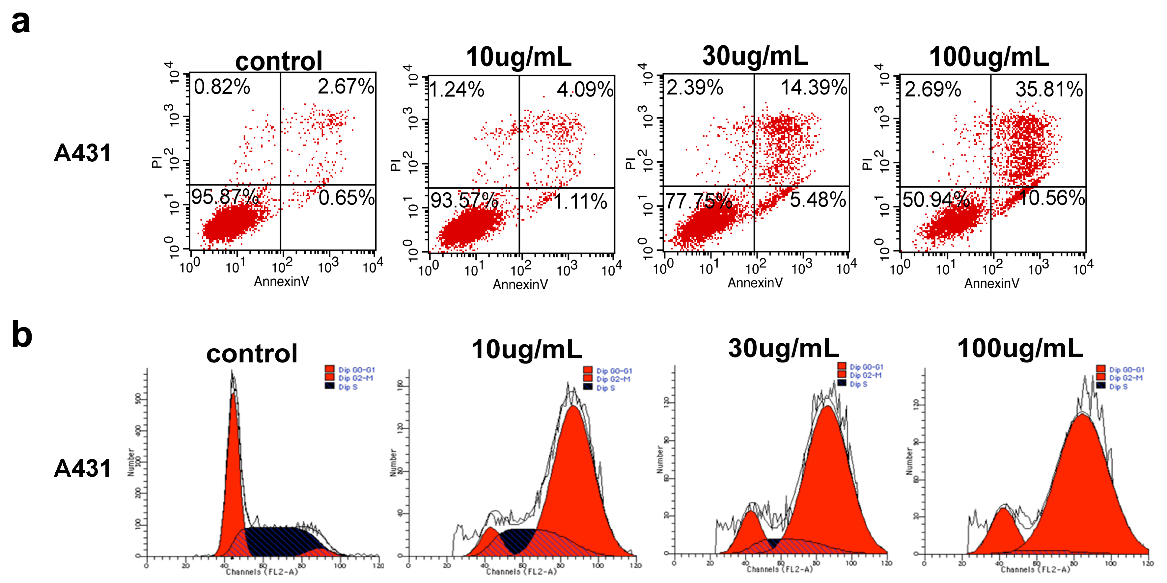


**Figure S5.** **Apoptosis and cell cycle arrest analysis in A431 cells by flow cytometry.** (a) The flow cytometry analysis of apoptosis in KYSE150 cells treated with various concentrations of LR004-VC-MMAE for 24 h**.** (b) The flow cytometry analysis of cell cycle arrest KYSE150 cells treated with various concentrations of LR004-VC-MMAE for 24 h.

**
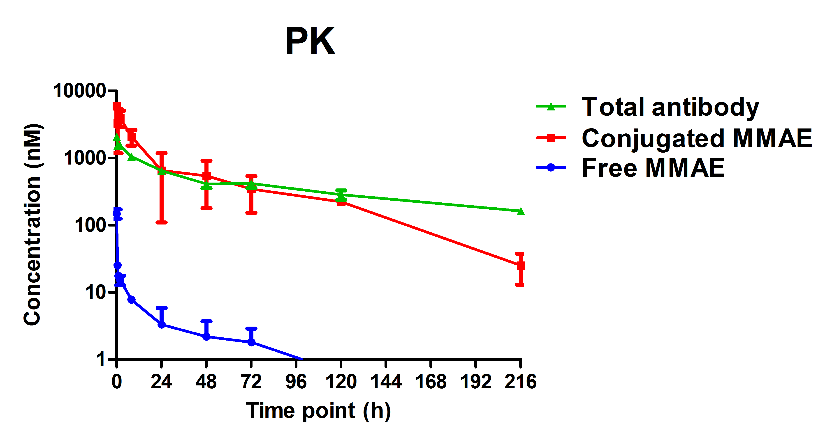
**

**Figure S6. PK profile of total antibody, conjugated MMAE and free MMAE administrated with LR004-VC-MMAE in nude mice model.** The BALB/c nude mice were injected subcutaneously with 15 mg/kg of LR004-VC-MMAE, and then sacrificed at 0 h, 0.5 h, 1 h, 2 h, 6 h, 24 h, 48 h, 72 h, 120 h and 216 h after serum collection. The concentration units was expressed as nM. Conjugated drug (red) started at higher concentration than Tab, and then decreased more rapidly than total antibody (green). The concentrations of free MMAE (blue) very low and decline over time.

**Table S1. Pharmacokinetic parameters of total antibody in nude mice**

| **pharmacokinetic parameters** | **Mean** | **SD** |
| --- | --- | --- |
| **Half-life (t_1/2_, h)** | 113.61 | 20.07 |
| **C_max_ (µg/mL)** | 318.2 | 19.4 |
| **AUC_0-t_ (h·µg/mL)** | 13069.2 | 1030.5 |
| **AUC_0-∞_ (h·µg/mL)** | 17087.9 | 883.9 |
| **MRT_0-t_ (h)** | 71.50 | 1.62 |
| **V_d_ (L/kg)** | 0.14 | 0.03 |
| **CL (mL/h/kg)** | 0.88 | 0.05 |

**Table S2. Pharmacokinetic parameters of conjugated MMAE in nude mice**

| **pharmacokinetic parameters** | **Mean** | **SD** |
| --- | --- | --- |
| **Half-life (t_1/2_, h)** | 33.31 | 6.15 |
| **C_max_ (ng/mL)** | 4288.8 | 426.5 |
| **AUC_0-t_ (h·ng/mL)** | 70983.3 | 10099.1 |
| **AUC_0-∞_ (h·ng/mL)** | 71929.1 | 10470.1 |
| **MRT_0-t_ (h)** | 43.02 | 2.54 |
| **V_d_ (L/kg)** | 0.17 | 0.02 |
| **CL (L/h/kg)** | 3.67 | 0.50 |

**Table S3. Pharmacokinetic parameters of free MMAE in nude mice**

| **pharmacokinetic parameters** | **Mean** | **SD** |
| --- | --- | --- |
| **Half-life (t_1/2_, h)** | 37.97 | 21.53 |
| **C_max_ (ng/mL)** | 106.4 | 16.5 |
| **AUC_0-t_ (h·ng/mL)** | 301.8 | 83.3 |
| **AUC_0-∞_ (h·ng/mL)** | 312.7 | 86.8 |
| **MRT_0-t_ (h)** | 32.69 | 7.48 |
| **V_d_ (L/kg)** | 43.99 | 14.03 |
| **CL (L/h/kg)** | 890.14 | 287.72 |
